# In Vitro Biological Activities of Fruits and Leaves of *Elaeagnus multiflora* Thunb. and Their Isoprenoids and Polyphenolics Profile

**DOI:** 10.3390/antiox9050436

**Published:** 2020-05-17

**Authors:** Sabina Lachowicz, Ireneusz Kapusta, Michał Świeca, Carla M. Stinco, Antonio J. Meléndez-Martínez, Anna Bieniek

**Affiliations:** 1 Department of Fermentation and Cereals Technology, Wrocław University of Environmental and Life Science, Chełmońskiego 37, 51-630 Wroclaw, Poland; 2 Department of Food Technology and Human Nutrition, Faculty of Biology and Agriculture, Rzeszow University, Zelwerowicza 4, 35-601 Rzeszow, Poland; ikapustai@ur.edu.pl; 3 Department of Biochemistry and Food Chemistry, University of Life Sciences in Lublin Skromna 8, 20-704 Lublin, Poland; michal.swieca@up.lublin.pl; 4 Food Colour & Quality Laboratory, Area of Nutrition & Food Science, Universidad de Sevilla, 41012 Seville, Spain; cstinco@us.es (C.M.S.); ajmelendez@us.es (A.J.M.-M.); 5 Department of Horticulture, University of Warmia and Mazury, Prawocheńskiego 21, 10-720 Olsztyn, Poland; anna.bieniek@uwm.edu.pl

**Keywords:** bioactive compounds, carotenoids, (*all-E*)-lycopene, provitamin A, chlorophylls, cherry silverberry

## Abstract

The objective of this study was in-depth identification of carotenoids and polyphenolic compounds in leaves and fruits of *Elaeagnus multiflora* Thunb. An additional aim was to assay their antioxidant and in vitro biological activities (the ability to inhibit pancreatic lipase, α-amylase, and α-glucosidase activity) of two cultivars: ‘Sweet Scarlet’ and ‘Jahidka’. Study results showed the presence of 70 bioactive compounds, including 20 isoprenoids and 50 polyphenols. The profile of identified bioactive compounds had not been examined in this respect until now. The total carotenoid, chlorophyll, and polyphenol levels and antioxidant activity of the foliar samples were virtually identical in both cultivars and clearly higher relative to those in the fruits. On the other hand, the ability to inhibit pancreatic lipase, α-amylase, and α-glucosidase activity of the fruits was clearly higher as compared to the leaves. The highest amount of phenolic acids, flavonols, and polymeric procyanidins was in the ‘Sweet Scarlet’ for fruit and leaves, while the highest amount of chlorophylls and carotenoids was in the ‘Jahidka’. The inhibition of α-amylase, α-glucosidase, and pancreatic lipase activities appeared to be better correlated with the carotenoid content, which warrants further studies of the possible anti-diabetic and anti-obesity actions of the major carotenoids found in the fruits (lycopene, phytoene, and lutein). In addition, strong correlation between antioxidant activity and phenols of *E. multiflora* Thunb. components can be effective in removing reactive oxygen species. The results of our study show that both the fruits and leaves of *E. multiflora* Thunb. can be important for health promotion through the diet and for innovating in the industry of functional food and (nutri)cosmetics.

## 1. Introduction

The study of biodiversity in the context of food security and health promotion through the diet has been and will continue to be an important research field, especially in the challenging current scenario of population growth and the climate change. The promotion of neglected edible species is being advocated by Food and Agriculture Organization of the United Nations (FAO) for several reasons, including that they can enrich our diets (by providing nutritious foods), safeguard our agriculture (by increasing the number of crops to feed humanity and reduce the impact of pests and other vulnerabilities), beat climate change (as traditional cultivars are interesting for their climate-resistant properties), and boost the livelihoods of small-scale farmers and local producers, among many others [1].

The study of *Elaeagnus multiflora* Thunb (*Elaeagnaceae*), called cherry silverberry or gumi, is important in this context. This species is native to China, Korea, and Japan. Its fruits and leaves have been known and used in traditional Chinese medicine as a remedy for cough, wounds, diarrhea, and even cancer lesions. Fruits of this species, being suitable for direct consumption and processing, have been ascribed health-promoting properties [2,3]. Today, this species is grown in the eastern part of the United States and in Europe, including Poland, as not only an ornamental plant but also a plant usable for homemade processing [4,5]. As demonstrated in the study conducted by Bieniek et al. [4], this plant grows very well in the moderate climate of Poland and is characterized by the ease of cultivation and resistance to diseases. According to literature data [2,3], the genus *Elaeagnaceae* comprises from 70 to 80 species. Only the health-promoting compounds of a few of them have been studied, e.g., *E*. *angustifolia* L. and *E*. *umbellate* Thunb. [6,7]. In turn, *E*. *multiflora* Thunb. has not been thoroughly characterized to date. The available data [5] shows that both the fruits and leaves of this species contain compounds of dietary interest, e.g., organic acids including the major malic acid, which accounts for 55–60% of total acids, as well as fatty acids, ascorbic acid (at 15.13 mg/100 g fresh weight) and other vitamins, pectins, and mineral compounds [5,8]. Lee et al. [2] and Patel [8] confirmed the presence of secondary metabolites like phenolic acids (caffeic, chlorogenic, and *p*-formic acids), flavonols (quercetin and rutin), and the carotenoid lycopene. These compounds have been associated with numerous health-promoting properties [9].

Polyphenols are plant secondary metabolites that contribute to the nutritional and sensory quality of plants. They constitute a large group of bioactive substances that intervene in various biological actions of plants [9,10]. They act as, among others, phytoalexins, attractants of pollinators, antioxidants, compounds influencing pigmentation, and protection against UV radiation, pathogens, and predators [9]. What is more, they exhibit excellent food-preserving and -coloring properties and have been linked to a reduction of the risk of developing diverse disorders including atherosclerosis, cardiovascular diseases, brain dysfunction, metabolic diseases, and also carcinogenic lesions [9].

Carotenoids are versatile dietary compounds as, apart from their role as natural pigments and as precursors of retinoids with vitamin A activity of some of them, there is a large body of evidence indicating that they can exhibit health-promoting biological actions that can contribute to enhance immunity and reduce the risk of developing diverse diseases, including cardiovascular disease and cancer as well as bone, skin, or eye disorders. The mechanisms by which carotenoids can promote health are diverse, such as antioxidant actions (quenching, scavenging), enhancement of gap junctional intercellular communication, modulation of signaling pathways or immune function, and absorption of visible light (or UV in the case of the colorless carotenoids, phytoene and phytofluene), which may interact and result in effects such as antioxidant or anti-inflammatory effects [11]. In addition to their role in photoprotection, carotenoids are important contributors to the color of the skin together with melanin, oxyhemoglobin, and deoxyhemoglobin, and evidence is accumulating that they could provide other aesthetic benefits, hence, their importance in nutricosmetics is expanding [12]. Humans can obtain carotenoids from plant products, algae, animal foods, or other products such as additives or nutritional supplements, the main contributors to their intake being fruits and vegetables [11]. The bioprospection of neglected edible plants to search for rich sources of carotenoids is an important field that has resulted in the identification of interesting species for the provision of health-promoting carotenoids. Some examples are sastra (*Garcinia intermedia*) (for zeaxanthin and lutein), corozo (*Aiphanes aculeata*) or sapote (*Quararibea cordata*) (for zeaxanthin), or sarsaparilla (*Smilax aspera*) (for lycopene) [13,14].

Considering that there are very few works about the bioactive compounds’ profile in selected morphological parts of *E. multiflora* Thunb. and their biological activity [2,3,5] this study was aimed at profiling the isoprenoid and polyphenolic contents of the leaves and fruits of two cultivars of *E. multiflora* Thunb, namely ‘Jahidka’ and ‘Sweet Scarlet’. Additionally, the antioxidant as the ability to radical cation scavenging activity (ABTS), radical scavenging activity (DPPH) and reducing activity (FRAP) and in vitro biological activities as the ability to inhibit pancreatic lipase, α-amylase, and α-glucosidase activity of such materials was assessed. Given the versatility and importance of the compounds examined, the results of this study can be important to encourage the inclusion in the diet of *E. multiflora* Thunb. for health promotion and for the development of innovative products for health promotion or cosmetic purposes.

## 2. Materials and Methods

### 2.1. Chemicals

The isoprenoid extraction solvents (dichloromethane, methanol, acetone) were of analytical grade (VWR, Seattle, WA, USA). Methanol (MeOH) and methyl tert-butyl ether (MTBE) for chromatographic analyses were of HPLC (High Performance Liquid Chromatography) grade (Merck, Darmstadt, Germany). Purified water (NANOpure^®^ DIamondTM, Barnsted Inc. Dubuque, IO, USA) was used for UPLC (Ultra Performance Liquid Chromatography). Standards for chlorophylls, α-carotene, β-carotene, lutein, and lycopene were obtained from Sigma-Aldrich (Steinheim, Germany). Violaxanthin, neoxanthin, and phytoene standards were obtained as explained elsewhere [15]. Pheophytin standards were obtained from their respective chlorophylls by adding diluted HCl (0.1 mol) [16]. Standards for keampferol-3-*O*-glucose, quercetin-3-*O*-glucose, quercetin-3-*O*-rutinose, isorhamnetin-3-*O*-rutinose, gallic acid, quinic acid, *p*-coumaric acid, sinapic acid, ellagic acid, and phloretin were obtained from Sigma-Aldrich (Steinheim, Germany).

### 2.2. Materials

Fruits and leaves of *Elaeagnus multiflora* Thunb. cultivars, ‘Jahidka’ and ‘Sweet Scarlet’ (~5 kg per cultivar) were collected from Milanówek near Warsaw and the University of Warmia and Mazury in Olsztyn, Poland. The samples were collected at the optimum ripening time in 2019.

### 2.3. Determination of Polyphenols

For the extraction and determination of phenolic compounds, a protocol described before by Lachowicz et al. [17,18] was followed. The samples of fruits and leaves (1 g) were extracted with 10 mL of mixture grade methanol, ascorbic acid, and acetic acid. The extraction was performed twice by incubation under sonication (20 min). The samples were centrifuged (10 min. 19,000× *g*) and supernatants were filtered (Hydrophilic PTFE (polytetrafluoroethylene) 0.20 μm membrane). All extractions were carried out in triplicate.

Quantitative and qualitative identification of polyphenolic compounds was determined with the UPLC-PDA-ESI-MS/MS Waters ACQUITY system (Waters, Milford, MA, USA) (consisting of a binary pump manager, sample manager, column manager, photodiode array detector (PDA) and QTof mass spectrometer with electrospray ionization (ESI)) as described earlier by Lachowicz et al. [18]. The polyphenols were determined based on molecular mass (in the negative ion mode), MS/MS ions, UV-VIS spectrum, and available data [19,20,21,22,23,24,25,26,27,28,29]. The calibration curves were run at 360 nm for the standard keampferol-3-*O*-glucose, quercetin-3-*O*-glucose, quercetin-3-*O*-rutinose, and isorhamnetin-3-*O*-rutinose; at 320 nm for the standard of gallic acid, quinic acid, *p*-coumaric acid, and sinapic acid; and at 240 nm for the standard ellagic acid, phloretin, at concentrations ranging from 0.05–5 mg/mL (*r^2^* = 0.9999). All samples were obtained in triplicate and expressed as mg/100 g of dry matter (d.m.).

### 2.4. Analysis of Proanthocyanidins by Phloroglucinolysis

Analysis of proanthocyanidins of samples was performed as described by Lachowicz et al. [18]. Fruits and leaves (5 mg) were mixed methanol solution with methanolic HCl and were incubated (at 50 °C, 30 min). After that the vials were put in an ice bath and 0.6 mL of the reaction medium was added, diluting with 1.0 mL of sodium acetate buffer. The samples were centrifuged (10 min, 20,000× *g* at 4 °C).

Phloroglucinolysis was analyzed using the liquid chromatograph Waters (Waters, Milford, MA, USA), consisting of a diode array and scanning fluorescence detectors, column manager. The separation was carried out using Cadenza CD C18 column (75 mm × 4.6 mm, 3 μm) kept at 15 °C. For phloroglucinolysis investigation the following solvent system (mobile phase A (25 mL acetic acid and 975 mL water) and mobile phase B (acetonitrile)) was applied. The detection of fluorescence was recorded at emission wavelength 360 nm and excitation wavelength 278 nm. The calibration curves and quantification were evaluated using standards: (−)-epicatechin, (+)-catechin, (−)-epicatechin-phloroglucinol, and (+)-catechins-phloroglucinol. The degree of polymerization was analyzed by evaluating the molar ratio of all the flavan-3-ol units. All results were evaluated in triplicate and administered as mg/100 g dry matter (d.m.).

### 2.5. Isoprenoids’ Analysis

The extraction of isoprenoids (carotenoids and chlorophylls) was evaluated, according to Stinco et al. [30]. Approximately 50 mg of powder from the samples were used for the extractions. Subsequently, 1 mL of extracting solvent (dichloromethane/methanol/acetone, 50:25:25 *v*/*v*/*v*) was added. The mixture was vortexed 5 min at 3500 rpm and ultrasonicated (Ultrasons, JP Selecta, Barcelona, Spain) for 2 min. To promote phase separation, the sample was centrifuged (Microfuge 22R, Beckman Coulter, Madrid, Spain) at 18,000× *g* for 5 min at 4 °C. After recovering the colored fraction, the residue was extracted again with another aliquot of 1 mL dichloromethane. This procedure was repeated until the color exhaustion.

The Rapid Resolution Liquid Chromatography (RRLC) and Ultra Performance Liquid Chromatography (UPLC) analyses of isoprenoids compounds were carried out according to the validated method described by Stinco et al. [16].

### 2.6. In Vitro Assessment of Health-Promoting Capacities

#### 2.6.1. Extraction Procedure

The material of fruit and leaves (~1 g) were mixed with 20 mL of PBS (phosphate-buffered saline) buffer (pH 7.4) and incubated (1 h). After that, the material of fruit and leaves was centrifuged per 15 min and the supernatants and supernatants were filtered.

#### 2.6.2. Antiradical activity

ABTS radical activity (ABTS•+) method was carried out according to an earlier protocol described by Re et al. [31]. Briefly, after 6 min of reaction of sample (0.03 mL + 3 mL of ABTS solution) the material was analyzed at 734 nm. All material was evaluated in triplicate and administrated in mmol Trolox(TE)/g d.m.

#### 2.6.3. Antiradical Activity

The free radical (DPPH) method was carried out according to an earlier protocol described [32]. A diluted extract was mixed with DPPH• dissolved in ethanol and water, and after 10 min of reaction the absorbance was measured at 517 nm. All samples were obtained in triplicate and expressed in mmol TE/g d.m.

#### 2.6.4. Reducing Activity

The ferric reducing antioxidant activity (FRAP) method was carried out following the method described by Benzie and Strain [33]. In brief, 0.1 mL of sample was mixed with 0.9 mL of distilled water and 3 mL of ferric complex. After 10 min of reaction, the absorbance was measured at 593 nm. All samples were obtained in triplicate and expressed in mmol TE/g d.m.

#### 2.6.5. The α-Amylase Inhibitor Activity Assay

The α-amylase inhibitor (αA) activity was measured according to the method described by Jakubczyk et al. [34]. The αA activity was expressed as AIU (the amount of inhibitor that completely inhibited one enzyme unit)/mg of sample.

#### 2.6.6. The α-Glucosidase Inhibitor Activity Assay

The α-glucosidase inhibitor (αG) activity was measured according to the method described by Jakubczyk et al. [34]. The αG was administrated as AIU (the amount of inhibitor that completely inhibited one enzyme unit)/mg of sample.

#### 2.6.7. Pancreatic Lipase Inhibitor Activity Assay

Pancreatic lipase inhibitory activity was determined with *p*-nitrophenyl acetate (*p*NPA) as the substrate according to the test described by Jakubczyk et al. [34]. Pancreatic lipase inhibitor activity was expressed as LIU (the amount of inhibitor that completely inhibited one enzyme unit)/mg of sample.

### 2.7. Statistical Analysis

Statistical analysis such as one-way ANOVA (*p* < 0.05), and Duncan’s multiple range were analysed using Statistica 12.5 (StatSoft, Kraków, Poland).

## 3. Results and Discussion

### 3.1. Polyphenolics’ Profile

Qualitative and quantitative data about the phenolic profile of the two cultivars studied are summarized in Table 1 and Table 2. Tentative identification revealed a total of 50 phenolic compounds in the examined material, including three phenolic acids, one hydrolyzable tannin, one stilbene, and 45 flavonols; 16 compounds were detected in fruits and 38 compounds in leaves of *Elaeagnus multiflora* Thunb. To date, very scarce information was available on the polyphenolic profile of fruits and leaves of these cultivars. A few compounds, usually phenolic acids, that were not detected in this study (Table 1 and Table 2) have been previously reported [2,3].

Among the phenolic acids, two compounds with numbers 1 and 2 were identified, based on fragmentation patterns and comparison with literature data [23,35], as quinic acid (*m/z* (*m* stands for mass and *z* stands for charge number of ions) 191) and 3-*p*-coumaroylquinic acid (*m/z* 337). These compounds were identified only in the leaves of *E. multiflora* Thunb. In turn, the compound detected in leaves (no. 11) and in fruits (no. 2) was tentatively identified as sinapic acid-*O*-hexoside based on findings reported by Spínola et al. [26] for *E. umbellata* Thunb.

Based on literature data, di-galloyl-hexahydroxydiphenoyl(HHDP)-glucoside (at *m/z* 785) [29] belonging to the group of hydrolyzable tannins and 3′,5′-Di-C-*β*-d-glucosylphloretin [28] belonging to the group of stilbenes were identified in fruits of *E. multiflora* Thunb.

Among the 45 tentatively identified flavonols, 15 were detected in fruits and 37 in leaves of *E. multiflora* Thunb. Only three compounds (with nos. 3, 14–16 in fruits and nos. 18, 36–38 in leaves) with the same molecular weight occurred in both morphological parts. In addition, only 13 compounds (with nos. 3, 5, 8–10, 13, 15, 20, 30, 32, 33, 36–38 in leaves and nos. 9, 11, 12, 14–16 in fruits) were earlier described in the literature [17,19,20,21,22,23,24,25,26,27] and confirmed in our work. As it can be noticed, the polyphenolic profile identified in leaves and fruits of *E. multiflora* Thunb. did not match the typical profile described in the literature for fruits or leaves of this species. Thus, the fruits and leaves of *E. multiflora* Thunb. are valuable sources because they contain compounds that had earlier been determined in various valuable plant materials, including *Hippophae rhamnoides* L., *Oxycoccus, Tilia americana var. mexicana, Amelanchier alnifolia* Nutt., *Tilia flas, Elaegnus umbellata* Thunb., and *Scutellaria immaculata* [17,19,20,21,22,23,24,25,26,27].

However, of the 43 flavonols identified, 27 had not been previously determined in any plant material. In leaves, tentative identification revealed 21 compounds, including five compounds belonging to quercetins and 15 to kaempferol derivatives (or glycons). Compound no. 4 was tentatively identified based on the primary ion at *m/z* 757 and MS/MS fragmentation giving ions at *m/z* 595, 463, and 301 through the loss of two glycoside residues and one pentose residue, respectively. A deprotonated molecule at *m/z* 901 was identified for compound no. 6 based on the loss of a rhamnose residue (146 Da) and fragmentation peaks at *m/z* 755, 609, 447, and 285, and based on the rhamnose residue and two glycol residues. These compounds were tentatively identified as quercetin glycoside-pentoside-glycoside and kaempferol di-rhamnoside-di-glucoside, respectively. In turn, compound no. 7 was classified as quercetin pentoside-rutinoside based on the loss of a pentose residue (132 Da) at the primary peak of *m/z* 741, and losses of rhamnose and glycol residues for ions at *m/z* 609, 463, and 301. Compound nos. 12 and 14 were identified at the primary *m/z* ion of 887, however, they had various primary ions, which for the first compound was determined at *m/z* 285 and allowed identifying it as kaempferol pentoside-rhamnoside-rutinoside, and for the second compound at *m/z* 301, which allowed identifying it as quercetin rhamnoside-pentoside-rhamnoside. Compound no. 16 with the primary ion of *m/z* 739 lost the rhamnose residue, while its fragmentation revealed peaks at *m/z* 593 and 285 and the loss of rutinoside residue, which enabled its tentative identification as kaempferol rhamnoside-rutinoside. In turn, compound no. 17 was tentatively identified as kaempferol rhamnoside-pentoside-rutinoside at the primary ion of *m/z* 871 and its fragmentation at *m/z* 563 [M–H–Rha–Glc]^–^ and 285 [M–H–2Rha–Glc–Pen]^–^. Compound no. 18 was tentatively identified as kaempferol pentoside-rutinoside (*m/z* 725), with its primary ion at *m/z* 285. Based on the pseudomolecular ion at *m/z* 725 and fragmentation ions at *m/z* 417 and 285 and due to the loss of rhamnose and pentose residues, compound no. 19 was tentatively identified as kaempferol rhamnoside-pentoside. In turn, compound no. 21 was identified based on the loss of a rhamnose residue at the primary ion *m/z* 901 and the loss of two glycol residues and one rhamnose residue during MS/MS fragmentation resulting in peaks at *m/z* 755, 609, 447, and 285. Compound no. 22 was tentatively identified as quercetin-*O*-glucoside-*O*-pentoside based on the primary ion at *m/z* 595 and its fragmentation at *m/z* 463 and 301. In turn, compound nos. 23, 24, and 27 were tentatively identified for the primary ion of *m/z* 739 and fragmentation ions at *m/z* 593, 447, and 285 as kaempferol di-rhamnoside-hexoside. Compound no. 25 was tentatively identified as quercetin di-rhamnose based on the primary ion at *m/z* 593 through the loss of the rhamnose residue (146 Da) and its fragmentation to ions at *m/z* 447 and 301 and the loss of another rhamnose residue. Compound no. 26 was tentatively identified as kaempferol pentoside-rhamnoside-glucuronide based on its fragmentation at the primary ion of *m/z* 739 into ions: At *m/z* 563 through the loss of a glucuronide residue, at *m/z* 417 through the loss of an additional rhamnose residue, and at *m/z* 285 through the loss of a pentose residue. In the case of compound no. 28, the primary peak occurred at *m/z* 709 due to the loss of a pentose residue (132 Da) and its fragmentation revealed peaks at *m/z* 577, 431, and 285 due to the loss of two additional rhamnose residues. Therefore, this compound was tentatively identified as kaempferol pentoside-di-rhamnoside. Based on the primary ion at *m/z* 577 and fragmentation ions at *m/z* 431 and 285, compound no. 29 was tentatively identified as kaempferol di-rhamnose due to the loss of two rhamnose residues. Compound no. 31 was tentatively presented as kaempferol glucoside-glucuronide based on its primary ion at *m/z* 623 and MS/MS fragmentation ions at *m/z* 447 and 285. In addition, compound no. 34 with the primary peak at *m/z* 891, respectively, had an acyl group (42 Da) and was tentatively identified as acetylated kaempferol derivatives based on the primary peak at *m/z* 285.

In addition, seven compounds, including four quercetin derivatives, two kaempferol derivatives, and one isorhamnetin derivative were tentatively identified in fruit, based on the presence of primary peaks at *m/z* 301, 285, and 315. For this reason, compound no. 1 was tentatively presented as quercetin-rhamnoside-pentoside-rutinoside based on its primary ion appearing at *m/z* 887 due to the loss of a rhamnose residue (146 Da) and fragmentation peaks at *m/z* 609, 579, and 301 due to the loss of pentose, rhamnose, and hexose residues, respectively. Compound no. 3 showed the main peak at *m/z* 725 and fragmentation ions at *m/z* 597, 417, and 285, and was tentatively identified as kaempferol-pentoside-rutinoside. Compound no. 5 was tentatively identified as quercetin-pentoside-rutinoside based on the primary ion at *m/z* 741 and its MS/MS fragmentation giving peaks at *m/z* 609 and 433. In turn, compound no. 6 was tentatively presented as quercetin-3-*O*-rhamnose-7-*O*-pentoside based on its primary ion at *m/z* 595 and fragmentation ions at *m/z* 433 and 301. Compound no. 7 was classified based on the pseudomolecular ion at *m/z* 739 due to the loss of rhamnose residue, and fragmentation of this compound revealing peaks at *m/z* 539 and 285 due to the loss of deoxyhexose residues. In turn, compound no. 8 was fragmented into peaks at *m/z* 593, 447, and 301, and due to the loss of three rhamnose residues was tentatively identified as quercetin-tri-rhamnoside (at *m/z* 739). In addition, compound no. 13 was tentatively identified as isorhamnetin 3-*O*-(6″malonyl)-glucuronide-rhamnoside based on its primary ion at *m/z* 723 and its MS/MS fragmentation ions at *m/z* 491 and 315.

The polyphenolic content in fruits of ‘Sweet Scarlet’ cultivars reached 1388.63 mg/100 g d.m., and was 1.4 times higher than in the fruits of ‘Jahidka’ cultivars. In turn, in leaves, it reached 1501.46 mg/100 g d.m. in the case of ‘Sweet Scarlet’ cultivars and 1080.84 mg/100 g d.m. in the ‘Jahidka’ cultivars, which was ca. 1.3 times higher compared to the fruits of the same cultivars. Leaves turned out to be richer in phenolics compared to fruits due to the complexity of the biosynthesis process in plants, which is dependent on various factors, including environmental signals related to the protection against biotic and abiotic stress or developmental signals [17,36,37]. On the other hand, it is important to consider that the levels of secondary metabolites in plants are dependent on diverse factors (agronomic, climatic, genotypic) [38], hence the difficulty of meaningfully comparing them across different studies. As reported by Jaakola et al. [39], a higher content of polyphenolic compounds was identified in leaves of plants growing under intensive sunlight, which results in the enhanced gene expression coupled with phenolic biosynthesis. In turn, according to Lee et al. [3], the content of phenols in fruits of *E. multiflora* Thunb. from Korea reached 315 mg/100 g d.m. and was 2.9 and 4.0 times lower than the fruits of ‘Jahidka’ and ‘Sweet Scarlet’ cultivars growing in Poland. As shown by Spínola et al., [26], the content of polyphenols in fruits of *E. umbellata* Thunb. was at 556 mg/100 g d.m. and was 1.6 and 2.3 lower compared to fruits of ‘Jahidka’ and ‘Sweet Scarlet’ cultivars of *E. multiflora* Thunb. belonging to the same family. In turn, the content of phenols in leaves of ‘Jahidka’ and ‘Sweet Scarlet’ cultivars of *E. multiflora* Thunb. was 3.6 and 2.6 times lower compared to their content in the same morphological part of *E. umbellata* Thunb. In turn, fruits of *E. angustifolia* L. were, on average, 10.8 times poorer in polyphenolics than fruits of *E. multiflora* Thunb., whereas in leaves their content reached 10.28 mg gallic acid equivalents (GAE)/100 g fresh mass [40]. From this information it can be stated that, considering the published data, the fruits of *E. multiflora* Thunb. growing in Poland are richer in polyphenolic compounds compared to other fruits of the same family.

In the analyzed fruits, procyanidin polymers accounted for 94.8% > flavonols for 5% > phenolic acids for 0.2% of total phenols, whereas in leaves they were also the major group of compounds and accounted for 81.2% > flavonols for 18.5% > phenolic acids 0.2% of total phenols. However, according to literature data, these were the flavonols that represented the major group of phenols in fruits of *E. umbellata* Thunb. with their content reaching 78.8%. In contrast, ellagitannins turned out to be the prevailing class in leaves accounting for 56.8% of total phenols [26]. In the fruits of *E. multiflora* Thunb. growing in China, the predominating class of compounds was represented by flavan-3-ols (75% of total compounds) [3]. In addition, the major flavonol of fruits turned out to be kaempferol-deoxyhexoside-pentoside (30%), whereas in leaves, kaempferol di-rhamnoside-glucoside (59%). Among the acids, sinapic acid-*O*-hexoside predominated in both fruits and leaves and accounted for 100% and 53% of total acids, respectively. In turn, kaempferol-*O*-(coumaroyl)hexoside and bis- HHDP-*O*-hexoside were the major compounds in fruits and leaves of *E. umbellata* Thunb., with their contents reaching 37.2% and 26.7%, respectively. Procyanidin polymers are characterized by a higher degree of polymerization compared to flavan-3-ol dimers, while these compounds have earlier been detected in, e.g., fruits and leaves of cranberry, with the HPLC method [17]. It is noteworthy that the content of procyanidin polymers has never been determined in fruits from the family *E. multiflora* Thunb. before. According to Pei et al. [41], the procyanidin polymers’ content in fruits of *E. umbellata* Thunb. was 255 mg catechin equivalents (CE)/100 g d.m., and was on average 4 times lower than in the fruits of *E. multiflora* Thunb.

### 3.2. Isoprenoids’ Profile: Chlorophylls and Carotenoids

Carotenoids are associated to chlorophylls in the photosynthetic apparatuses of plants and other photosynthetic organisms, where they enhance the efficiency of light harvesting and electron transfer, protect against photooxidation, and intervene in their assembly and stabilization, with the amount of chlorophylls in these associations being higher [42,43]. Thus, the levels of chlorophylls were considerably higher relative to those of carotenoids. The levels of total chlorophylls were significantly different between the two cultivars (Table 3) [44]. The carotenoid profile of photosynthetic tissues is very well conserved. Normally, the major carotenoids are lutein, β-carotene, violaxanthin, and neoxanthin, in this order (the latter two at very similar levels). Lutein and the provitamin A carotenoid *β*-carotene are bioavailable in humans and among the most extensive carotenoids in relation to health promotion [45]. Minor carotenoids include *α*-carotene, *β*-cryptoxanthin, zeaxanthin, antheraxanthin, and lutein 5,6-epoxide. Lettuce photosynthetic tissues are a special case as they accumulate an unusual xanthophyll named lactucaxanthin [46]. Considering this paradigm, it can be readily inferred that the green leaves of the samples analyzed contained unusually elevated levels of α-carotene, which was, in fact, the second predominant carotenoid to lutein (Table 3). This fact can also be important from a nutritional point of view as α-carotene is a major provitamin A carotenoid, which is essential to fight vitamin-A deficiency in many regions [11]. Interestingly, the nutritional importance of the little-studied α-carotene is being pinpointed in studies carried out in the last decade. In relation to the major dietary provitamin A carotenoids, both α-carotene- and *β*-cryptoxanthin-rich foods are thought to exhibit greater apparent bioavailability than *β*-carotene-rich foods in Western diets [47]. The *α*-carotene circulating levels have been reported to be positively correlated with bone mineral density in Chinese individuals [48]. Furthermore, α-carotene has been shown to inhibit metastasis in Lewis lung carcinoma in vitro and to suppress lung metastasis and tumor growth (in association with taxol) in tumor-xenografted mice [49]. The intake of this carotenoid has also been shown to be associated to a reduced risk of prostate malignancy in Japanese [50].

Regarding the carotenoid profile of fruits, lycopene was the predominant carotenoid. This has also been extensively studied in relation to health promotion [45]. Lutein and phytoene were also detected. The colorless carotenoid, phytoene, has been largely neglected in food science and technology, nutrition, and health, although it is gaining momentum in research due to accumulating evidence indicating that it is a major dietary carotenoid, readily bioavailable in humans and that can intervene in biological actions that can be positive for health and even beauty promotion [12,51]. The total carotenoid levels of the foliar samples (calculated as the sum of the individual contents) were virtually identical, although significantly different. In the case of the fruits, there were clear differences as the levels in the ‘Jahidka’ cultivar were ~2.4-fold higher relative to those of the ‘Sweet Scarlet’ cultivar sample (971.48 and 400.93 mg/100 g d.m., respectively, data not shown). Statistically significant differences in the levels of lutein and lycopene were obtained. The levels of carotenoids in plants depend on factors of a different nature, including genotype, climatic conditions of the production area, or agronomic factors, among others [52].

### 3.3. In Vitro Assessment of Health-Promoting Capacities

#### 3.3.1. In Vitro Antioxidant Capacity

The antiradical activity assayed in leaves of ‘Sweet Scarlet’ and ‘Jahidka’ cultivars of *E. multiflora* Thunb. reached 5.56 and 5.08 mmol Trolox (TE)/g d.m. for ABTS assay and 2.69 and 2.51 mmol TE/g d.m. for DPPH assay, respectively (Table 4). These values were 9.7 and 6.2 times and 9.8 and 5.9 times higher than in the fruits of the respective cultivars. In turn, the reducing potential (FRAP assay) determined in leaves of ‘Sweet Scarlet’ and ‘Jahidka’ cultivars reached 0.30 and 0.33 mmol TE/g d.m., respectively, and was 3.7 and 2.6 times higher compared to the fruits of the same cultivars of cherry silverberry. As indicated by literature data and results of the present study, the value of the antioxidant capacity is strongly Pearson correlated with the content of bioactive compounds, including phenols (*r^2^* = 0.912) and carotenoids (*r^2^* = 0.539), which was confirmed by strong correlations [17,26]. In addition, as reported by Spínola et al. [26], leaves of *E. umbellate* Thunb. exhibited 6.9 times antiradical activity compared to fruits, which was consistent with our observations. Furthermore, leaves and fruits of *E. umbellate* showed 1.2 and 1.4 times lower activity, respectively, compared to the same morphological parts of *E. multiflora* Thunb. The values of antiradical activity obtained for fruits and leaves of cranberry, bilberry, and quince [36] were 7.0 and 5.3 times, 1.7 and 6.6 times, and 8.9 and 4.4 times lower, respectively, compared to fruits and leaves of *E. multiflora* Thunb. In turn, the reducing potential determined for fruits and leaves of cranberry [36] was similar to the results obtained for *E. multiflora* Thunb., whereas its value assayed for fruits and leaves of bilberry and quince [36] was, respectively, 2.8 and 1.8 times as well as 2.0 and 2.2 times higher compared to the same morphological parts of *E. multiflora* Thunb. It is worthy of mention that the antiradical activity of *E. multiflora* Thunb. leaves was similar to the activity determined for green tea [53], which indicates a high potential of not only fruits but also leaves of fruit trees, including leaves of *E. multiflora* Thunb., as ingredients in developing tea formulas or as additives in designing functional foods safe to consumers. In their study into the toxicity of cranberry leaves, Booth et al. [54] demonstrated that their extract could be used in the production of functional food safe to consumers.

#### 3.3.2. The Ability to Inhibit α-Amylase, α-Glucosidase, and Pancreatic Lipase Activity

Fruits and leaves of *Elaeagnus multiflora* Thunb. were also analyzed as potential sources of substances inhibiting activities of enzymes involved in the pathogenesis of the metabolic syndrome (Table 4). The against inhibition of α-amylase (αA) and α-glucosidase (αG) activities in cherry silverberry fruits of ‘Jahidka’ and ‘Sweet Scarlet’ cultivars showed no statistically significant differences, whereas leaves of ‘Jahidka’ cultivars were 20.6 and 1.7 times, and leaves of ‘Sweet Scarlet’ cultivars were 7.7 and 2.1 times less active than the fruits of the same cultivars. The aforementioned enzymes take part in the degradation of complex saccharides to become absorbable during digestion, whereas inhibition of their activity is used in the prevention of type 2 diabetes [50,52]. A similar extent of αG activity inhibition was obtained in the case of leaves of *E. angustifolia* L. [53]. The study conducted by Spínola et al. [26] demonstrated that the inhibition of αA and αG activities determined in *E. umbellata* Thunb. reached the biochemical half maximal inhibitory concentration (IC_50_) = 4.01 and 6.02 mg/mL in fruits as well as IC_50_ = 2.18 and 4.76 mg/mL in leaves, respectively, whereas in *Sambucus lanceolata* it reached IC_50_ = 6.01 and 7.55 mg/mL in fruits as well as IC_50_ = 7.71 and 4.97 mg/mL in leaves, respectively. In that study, however, results were provided as IC_50_ values, which makes their comparison with our data impossible. Nevertheless, the above authors noted that the leaves exhibited more inferior ability to inhibit activities of the analyzed enzymes, which was consistent with our observations. In addition, they showed a weak Pearson’s correlation with the total content of phenols.

In turn, the inhibition of pancreatic lipase activity by fruits of ‘Jahidka’ cultivars reached 1.39 U/g dry matter and was 1.9 times higher compared to ‘Sweet Scarlet’ cultivars, whereas no pancreatic lipase-inhibiting activity was demonstrated in leaves (Table 4). This enzyme is responsible for the breakdown of lipids to become absorbable in the intestinal lumen [55]. Inhibition of its activity is exploited in obesity prevention [26]. As reported by Spínola et al. [26], the inhibition of pancreatic lipase activity determined in *E. umbellate* Thunb. and *S. lanceolate* reached IC_50_ = 9.68 and 7.75 mg/mL in fruits, respectively, and turned out to be 1.9 and 1.2 times lower in leaves, respectively. These authors demonstrated also that the higher capability for inhibiting its activity in *S. lanceolate* was strongly correlated with the phenolic profile, including mainly anthocyanins, claimed to be its inhibitors, which were however not identified in *E. umbellate* Thunb. Other reported inhibitors of pancreatic lipase activity include ellagitannins and 5-*O*-coumarylquinic acid [26,56]. This can be the cause of the incapability for inhibiting this enzyme activity in leaves of *Elaeagnus multiflora* Thunb., characterized by a diverse phenolic profile. What is more, the results showed a strong Pearson’s correlation with the total content of carotenoids with the inhibition of pancreatic lipase activity *r^2^* = 0.938, while the correlation between the inhibition of pancreatic lipase activity and sum of polyphenols was positive *r^2^* = 0.307. The higher correlation with carotenoids is due to the fact that adipose tissue is a main organ in obesity etiology and site of storage for carotenoids [57].

According to literature data [26,58,59], hyperglycemia enhances the generation of reactive oxygen species that may lead to the development of inflammatory conditions and carcinogenic lesions. However, it turns out that both leaves and fruits exhibit high antioxidant activities in in vitro biological activities due to the abundance of their bioactive compounds [60], which are capable of not only inducing hypoglycemia but also removing reactive oxygen species [26].

As far as the inhibition of the three enzymes’ activities tested was concerned, it was observed that the correlations between the levels of phenolics and the ability to inhibit αA, αG, and pancreatic lipase were *r^2^* = 0.647, *r^2^* = 0.350, and *r^2^* = 0.307, respectively. In the case of carotenoids, the correlations were *r^2^* = 0.996, *r^2^* = 0.959, and *r^2^* = 0.938, respectively. These results suggested that the inhibiting activities were more related to the carotenoid levels than to the phenolic levels. Furthermore, our results suggest that the total phenolics’ content was less important in the inhibition of the analyzed enzymes compared to contents of particular classes of phenols [25,61]. A large diversity of structures of phenols or carotenoids within a class or between various classes of these compounds determines their capability to bind with digestive enzymes [26,59,62]. This may be the reason for differences in the ability to inhibit enzymatic activity by leaves and fruits of *E.s multiflora* Thunb. Besides, phenols in plant extracts can exhibit the antagonistic and/or synergistic activity towards digestive enzymes (the main enzymes digestive of carbohydrates are α-amylase and α-glucosidase, and of fats it is pancreatic lipase) and influence the inhibitory activities [26,62,63]. Reported inhibitors of αG activity of *E. umbellata* Thunb. include coumaroylquinic acid and proanthocyanidins, while proanthocyanidins, rutin, and quercetin were the main compounds of *E. umbellata* Thunb. [26] and also occurred in *E. multiflora* Thunb. against to inhibit α-amylase.

## 4. Conclusions

The leaves were found to contain 13 isoprenoids with chlorophyll found as the major compound, and 38 phenolic compounds with kaempferol di-rhamnoside-glucoside and sinapic acid-*O*-hexoside as the predominating ones. In fruits, eight isoprenoids were detected, including (*all*-E)-lycopene as the major compound, and also 16 phenolics including kaempferol-deoxyhexoside-pentoside and sinapic acid-*O*-hexoside as the major compounds. However, among the identified classes of phenolic compounds, the procyanidin polymers were found to prevail as they accounted for 94.8% and 81.2% of total phenols in fruits and leaves of *E multiflora* Thunb., respectively.

The total carotenoid levels of the foliar samples were virtually identical in both cultivars and clearly higher relative to those in the fruits. Apart from the health-promoting carotenoids, lutein and β-carotene, the leaves of the *E multiflora* Thunb. samples analyzed contained unusually high levels of α-carotene, a provitamin A carotenoid that may be important to promote bone health and to decrease the risk of cancer. The fruits contain lycopene as the predominant carotenoid as well as low levels of lutein and phytoene, all being of interest in terms of health promotion. On the other hand, the study of the unusually high levels of α-carotene in leaves in the context of photosynthesis appears as an interesting topic for further studies, as the carotenoid profile of plant photosynthetic tissues is normally very constant and well conserved. Leaves also turned out to be richer in phenolics compared to fruits. The polyphenolic content in leaves and fruits of ‘Sweet Scarlet’ were ~1.5- and 1.4-fold higher relative to those of the ‘Jahidka’ cultivar. In addition, the antiradical capacity and reduction potential were found to be higher in the leaves than in the fruits and also showed strong correlation with polyphenols that may be important to remove reactive oxygen species, and thus prevent the promotion of cancer.

The against inhibition of αA and αG activities of the fruits was similar in both cultivars, and clearly higher as compared to the leaves. The inhibition of pancreatic lipase activity was only detected in fruits and it was ~1.9-fold higher in the ‘Jahidka’ cultivars. The inhibition of the analyzed enzymes’ capacities appeared to be better correlated with the carotenoid content, which warrants further studies of the possible anti-diabetic and anti-obesity actions of lycopene, phytoene, and lutein.

In summary, the results of this study indicate that the inclusion of fruits and leaves on *E. multifolia* Thunb. in the diet is desirable in order to increase the intake of vitamin A (in the form of α- and β-carotene) and polyphenolic compounds that could contribute to promote health by diverse mechanisms and also may be effective in the prevention of chronic noncommunicable diseases. Such materials also appear as promising in order to innovate in the context of functional foods and other industries, including (nutri)cosmetics.

## Figures and Tables

**Table 1:** Summary of qualitative and quantitative data of the polyphenolic profile in leaves of *Elaeagnus multiflora* Thunb.

Peak No.	Tentative Identification Compounds	Rt (min)	MS (*m/z*)	Fragment Ion (*m/z*)	UV-Vis (nm)	Jahidka	Sweet Scarlet
**Phenolic acids**							
1	Quinic acid	1.05	191	172	262	0.48 ± 0.06 ^a^	0.85 ± 0.01 ^a^
2	3-*p*-coumaroylquinic acid	1.33	337	191	276	0.44 ± 0.09 ^b^	1.38 ± 0.11 ^a^
11	Sinapic acid-*O*-glucoside	3.36	385	223	325	2.25 ± 0.18 ^a^	1.23 ± 0.11 ^b^
Σ Phenolic acid						3.18 ± 0.04 ^a^	3.45 ± 0.22 ^a^
Flavonols							
3	Methyl-quercetin 3-*O*-rhamnoside-pentoside	1.94	609	463/331/299	317	0.26 ± 0.03 ^a^	0.28 ± 0.01 ^a^
4	Quercetin glycoside-pentoside-glycoside	2.53	757	595/463/301	255/352	2.68 ± 0.03 ^a^	1.79 ± 0.16 ^b^
5	Kaempferol 3-*O*-rutinoside-7-*O*-glucoside	2.58	755	609/447/285	267/350	4.13 ± 0.50 ^a^	0.99 ± 0.01 ^b^
6	Kaempferol di-rhamnoside-di-glucoside	2.78	901	755/609/447/285	266/350	0.66 ± 0.06 ^b^	1.33 ± 0.14 ^a^
7	Quercetin pentoside-rutinoside	2.8	741	609/463/301	255/350	2.67 ± 0.11 ^a^	1.11 ± 0.01 ^b^
8	Kaempferol 7-*O*-pentoside	2.87	417	285	281/340	0.56 ± 0.01 ^a^	0.82 ± 0.07 ^a^
9	Kaempferol 3-*O*-rhamnoside	3.14	431	285	267/325	1.50 ± 0.10 ^a^	0.69 ± 0.03 ^b^
10	Kaempferol glucoside-rutinoside	3.17	755	609/285	266/319	4.62 ± 0.02 ^a^	0.63 ± 0.01 ^b^
12	Kaempferol pentoside-rhamnoside-rutinoside	3.43	887	755/609/285	264/338	1.74 ± 0.08 ^b^	5.38 ± 0.20 ^a^
13	Quercetin 3-*O*-rutinoside	3.44	609	301	255/352	0.97 ± 0.09 ^b^	2.03 ± 0.10 ^a^
14	Quercetin rhamnoside-pentoside-rhamnoside	5.45	887	579/301	255/355	6.73 ± 0.01 ^a^	1.72 ± 0.11 ^b^
15	Quercetin 3-*O*-rhamnoside	3.49	447	301	255/326	5.57 ± 0.60 ^a^	1.33 ± 0.02 ^b^
16	Kaempferol rhamnoside-rutinoside	3.53	739	593/285	265/326	4.47 ± 0.01 ^b^	10.91 ± 0.44 ^a^
17	Kaempferol rhamnoside-pentoside-rutinoside	3.66	871	563/285	266/340	2.79 ± 0.25 ^a^	3.72 ± 0.20 ^a^
18	Kaempferol pentoside-rutinoside	3.71	725	593//285	265/345	2.71 ± 0.11 ^b^	6.61 ± 0.21 ^a^
19	Kaempferol rhamnoside-pentoside	3.79	563	417/285	265/328	1.14 ± 0.03 ^a^	0.60 ± 0.07 ^b^
20	Kaempferol 3-*O*-rutinoside	3.84	593	447/285	265/334	6.30 ± 0.57 ^a^	3.91 ± 0.17 ^b^
21	Kaempferol di-rhamnoside-di-glycoside	3.97	901	755/609/447/285	264/339	1.07 ± 0.04 ^a^	0.16 ± 0.01 ^b^
22	Quercetin-*O*-glucoside-*O*-pentoside	4.16	595	463/301	255/345	5.48 ± 0.27 ^b^	7.52 ± 0.21 ^a^
23	Kaempferol di-rhamnoside-glucoside	4.19	739	593/447/285	264/345	120.20 ± 7.83 ^b^	187.06 ± 29.38 ^a^
24	Kaempferol di-rhamnoside-glucoside	4.37	739	593/447/285	265/338	3.00 ± 0.04 ^a^	3.06 ± 0.04 ^a^
25	Quercetin di-rhamnose	4.47	593	447/301	255/347	4.75 ± 0.19 ^b^	13.04 ± 1.04 ^a^
26	Kaempferol pentoside-rhamnoside-glucuronide	4.59	739	563/417/285	265/324	2.22 ± 0.12 ^b^	3.74 ± 0.34 ^a^
27	Kaempferol di-rhamnoside-hexoside	4.66	739	593/447/285	265/319	1.57 ± 0.05 ^a^	1.28 ± 0.18 ^a^
28	Kaempferol pentoside-di-rhamnoside	4.8	709	577/431/285	265/339	9.91 ± 1.14 ^a^	5.19 ± 0.47 ^b^
29	Kaempferol di-rhamnose	4.89	577	431/285	264/341	10.90 ± 0.47 ^b^	29.15 ± 0.85 ^a^
30	Kaempferol-3-*O*-glucoside	5.01	447	285	264/319	1.08 ± 0.07 ^b^	1.38 ± 0.23 ^a^
31	Kaempferol glucoside-glucuronide	5.12	623	447/285	264/317	0.31 ± 0.01 ^b^	0.71 ± 0.03 ^a^
32	Eriodictyol glucoside-pentoside	5.19	581	287	265/315	2.13 ± 0.33 ^a^	0.84 ± 0.06 ^b^
33	Kaempferol malonyl-glucuronide	5.27	547	461/285	265/315	2.38 ± 0.03 ^a^	0.97 ± 0.05 ^b^
34	Unknown derivatives of Kaempferol	5.63	891	285	269/325	0.64 ± 0.05 ^a^	0.01 ± 0.00 ^b^
35	Kaempferol 3-*O*-rhamnoside	5.94	431	285	264/325	0.21 ± 0.02 ^a^	0.26 ± 0.04 ^a^
36	Kaempferol 3-*O*-(6″-*p*-coumaryl)-galactoside	6.87	593	447/285	267/312	0.34 ± 0.05 ^a^	0.99 ± 0.15 ^a^
37	Kaempferol 3-*O*-(6″-caffeoyl)-glucoside	7	623	447/285	264/321	0.46 ± 0.04 ^b^	1.17 ± 0.07 ^a^
38	Kaempferol 3-*O*-(6″-*p*-coumaryl)-glucoside	7.11	593	447/285	267/315	0.14 ± 0.01 ^b^	0.29 ± 0.03 ^a^
Σ Flavonols						216.30 ± 9.80 ^b^	300.67 ± 31.19 ^a^
Polymeric procyanidins						947.50 ± 2.31 ^b^	1317 ± 3.91 ^a^
Degree of polymerization						6.20 ^a^	8.62 ^a^
Σ Phenolic compounds						1166.98 ^b^	1621.12 ^a^

Values are expressed as the mean (*n* = 3) ± standard deviation and different letters (between cultivars) within the same row indicate statistically significant differences (*p* < 0.05).

**Table 2:** Summary of qualitative and quantitative data of the polyphenolic profile in fruits of *E. multiflora* Thunb.

Peak No.	Tentative Identification Compounds	Rt (min)	[M–H]^–^ (*m/z*)	Fragment Ions (*m/z*)	UV-Vis (nm)	Jahidka	Sweet Scarlet
Phenolic acids							
2	Sinapic acid-*O*-glucoside	3.53	385	223	300	1.22 ± 0.24 ^b^	3.80 ± 0.19 ^a^
Flavonols							
1	Quercetin-rhamnoside-pentoside-rutinoside	3.39	887	609/579/301	255/352	1.40 ± 0.17 ^b^	1.38 ± 0.11 ^a^
3	Kaempferol-pentoside-rutinoside	3.67	725	579/417/285	267/350	6.58 ± 0.71 ^b^	27.45 ± 2.50 ^a^
5	Quercetin-pentoside-rutinoside	3.91	741	609/433	255/350	5.08 ± 0.62 ^b^	10.73 ± 1.15 ^a^
6	Quercetin-3-*O*-rhamnoside-7-*O*-pentoside	4.12	595	433/301	255/350	1.56 ± 0.14 ^b^	2.14 ± 0.01 ^a^
7	Kaempferol-rhamnoside-rutinoside	4.24	739	593/285	265/340	0.87 ± 0.03 ^a^	1.33 ± 0.12 ^a^
8	Quercetin-tri-rhamnoside	4.33	739	593/447/301	255/355	5.19 ± 0.02 ^a^	2.79 ± 0.12 ^b^
9	Quercetin-rhamnoside-glucopyranoside-rhamnoside	4.48	769	593/447/301	255/352	3.22 ± 0.21 ^a^	1.85 ± 0.04 ^b^
11	Isorhamnetin-7-*O*-rutinoside	5.09	623	477/315	253/358	6.03 ± 0.49 ^a^	3.09 ± 0.11 ^b^
12	Isorhamnetin-3-*O*-glucoside	5.20	477	315	256/380	0.95 ± 0.05 ^a^	0.18 ± 0.01 ^b^
13	Isorhamnetin 3-*O*-(6″malonyl)-glucuronide-rhamnoside	5.71	723	491/315	270/350	0.85 ± 0.08 ^b^	1.39 ± 0.09 ^a^
14	Kaempferol-3-*O*-(6″-*p*-coumaryl)-glucoside	6.85	593	285	267/312	0.75 ± 0.01 ^a^	0.09 ± 0.01 ^b^
15	Kaempferol 3-*O*-(6′-caffeoyl)-glucoside	7.04	623	447/285	269/350	1.55 ± 0.17 ^a^	0.21 ± 0.01 ^b^
16	Kaempferol-3-*O*-(6″-*p*-coumaryl)-glucoside	7.10	593	285	267/330	1.00 ± 0.01 ^a^	1.25 ± 0.07 ^a^
Σ Flavonols						37.29 ± 0.86 ^b^	56.25 ± 3.93 ^a^
Hydrolyzable tannins							
4	Di-galloyl-HHDP-glucoside	3.73	785	633/483	271	3.07 ± 0.04 ^b^	10.60 ± 0.02 ^a^
Stilbenes							
10	3′,5′-Di-C-*β-D*-glucosylphloretin		597	579/507/477	285	1.71 ± 0.01 ^a^	0.91 ± 0.08 ^b^
Polymeric procyanidins						861.36 ± 6.57 ^b^	1197.34 ± 8.31 ^a^
Degree of polymerization						11.78 ^b^	16.37 ^a^
Σ Polyphenolic compounds						904.65 ^b^	1268.90 ^a^

Values are expressed as the mean (*n* = 3) ± standard deviation and different letters (between cultivars) within the same row indicate statistically significant differences (*p* < 0.05).

**Table 3:** Quantification of carotenoids and chlorophylls (mg/100 g dry matter (d.m.)) in leaves and fruits of *Elaeagnus multiflora* Thunb.

Leaves		
Compounds	Sweet Scarlet	Jahidka
Chlorophylls		
chlorophyll a- d1	94.35 ± 0.92 ^a^	54.97 ± 2.90 ^b^
chlorophyll b	427.99 ± 1.75 ^a^	408.64 ± 10.43 ^b^
chlorophyll a	1116.75 ± 4.57 ^a^	1096.53 ± 19.80 ^a^
chlorophyll a- d2	36.83 ± 1.52 ^a^	33.53 ± 2.15 ^a^
pheophytin a	18.23 ± 0.11 ^a^	40.51 ± 2.23 ^b^
Σ Chlorophylls	1694.16 ± 7.92 ^a^	1634.18 ± 35.24 ^b^
Carotenoids		
*Z*-violaxanthin	11.29 ± 0.49 ^a^	10.64 ± 0.43 ^a^
neoxanthin	23.78 ± 0.69 ^a^	22.98 ± 1.59 ^a^
violaxanthin	10.11 ± 0.13 ^a^	8.05 ± 0.31 ^b^
lutein	87.63 ± 0.95 ^a^	86.53 ± 2.50 ^a^
α-carotene	58.98 ± 0.87 ^a^	63.65 ± 1.35 ^b^
(9*Z*)-α-carotene	7.22 ± 0.35 ^a^	7.61 ± 0.42 ^a^
β-carotene	33.04 ± 0.23 ^a^	37.03 ± 1.69 ^b^
(9*Z*)-β-carotene	4.60 ± 0.03 ^a^	4.95 ± 0.20 ^b^
∑α-carotene isomers	66.20 ± 1.22 ^a^	71.26 ± 1.68 ^b^
∑β-carotene isomers	37.64 ± 0.21 ^a^	41.98 ± 1.88 ^b^
Σ Carotenoids	236.64 ± 3.50 ^a^	241.45 ± 8.11 ^a^
**Fruits**		
**Compounds**	**Sweet Scarlet**	**Jahidka**
Carotenoids		
phytoene	0.97 ± 0.06 ^a^	0.93 ± 0.06 ^a^
lutein	0.08 ± 0.01 ^a^	0.14 ± 0.01 ^b^
(15*Z*)-lycopene	0.14 ± 0.02 ^a^	0.19 ± 0.02 ^b^
(13*Z*)-lycopene	0.69 ± 0.05 ^a^	1.21 ± 0.07 ^b^
di-*Z* lycopene	0.43 ± 0.03 ^a^	0.78 ± 0.03 ^b^
(9*Z*)-lycopene	0.33 ± 0.02 ^a^	0.63 ± 0.03 ^b^
(all-*E*)-lycopene	35.23 ± 0.73 ^a^	87.70 ± 2.34 ^b^
(5*Z*)-lycopene	2.22 ± 0.04 ^a^	5.56 ± 0.19 ^b^
∑ lycopene isomers	39.04 ± 0.83 ^a^	96.08 ± 2.65 ^b^
Σ Carotenoids	40.09 ± 0.85 ^a^	97.15 ± 2.71 ^b^

Values are expressed as the mean (*n* = 3) ± standard deviation and different letters (between cultivars) within the same row indicate statistically significant differences (*p* < 0.05).

**Table 4:** Summary of the results obtained for the antioxidant and in vitro biological activities carried out with fruit and leaves of *Elaeagnus multiflora* Thunb.

Sample	Cultivar	Inhibition of α-Amylase Activity (U/g)	Inhibition of α-Glucosidase Activity (U/g)	Inhibition of Pancreatic Lipase Activity (U/g)	ABTS (mmolTE/g d.m.)	FRAP (mmolTE/g d.m.)	DPPH (mmolTE/g d.m.)
Fruits	Jahidka	101.16 ± 4.46 ^a^	14.99 ± 0.07 ^a^	1.39 ± 0.17 ^a^	0.90 ± 0.01 ^a^	0.13 ± 0.01 ^a^	0.44 ± 0.02 ^a^
	Sweet Scarlet	104.83 ± 0.61 ^a^	13.34 ± 0.19 ^a^	0.75 ± 0.07 ^b^	0.52 ± 0.01 ^b^	0.05 ± 0.01 ^b^	0.26 ± 0.01 ^b^
Leaves	Jahidka	4.91 ± 0.18 ^b^	8.73 ± 0.52 ^a^	-	5.08 ± 0.05 ^b^	0.30 ± 0.01 ^a^	2.59 ± 0.03 ^b^
	Sweet Scarlet	13.59 ± 0.15 ^a^	6.48 ± 0.51 ^b^	-	5.56 ± 0.05 ^a^	0.33 ± 0.01 ^a^	2.61 ± 0.03 ^a^

Values are expressed as the mean (*n* = 3) ± standard deviation and different letters (between cultivars) within the same row indicate statistically significant differences (*p* < 0.05).
